# Insights into 3-hydroxypropionic acid biosynthesis revealed by overexpressing native glycerol dehydrogenase in *Klebsiella pneumoniae*

**DOI:** 10.1080/13102818.2014.944419

**Published:** 2014-09-23

**Authors:** Ming-Yue Su, Ying Li, Xi-Zhen Ge, Ping-Fang Tian

**Affiliations:** ^a^ College of Life Science and Technology, Beijing Key Laboratory of Bioprocess, Beijing University of Chemical Technology, Beijing, P. R. China; ^b^ College of Biochemical Engineering, Beijing Union University, Beijing, P. R. China

**Keywords:** 3-Hydroxypropionic acid, glycerol dehydrogenase, *Klebsiella pneumoniae*, overexpression

## Abstract

In *Klebsiella pneumoniae*, glycerol dissimilation involves parallel oxidation and reduction pathways. Oxidation pathway provides adenosine triphosphate (ATP) and cofactors to sustain cell growth, while reduction pathway presents 3-hydroxypropionic acid (3-HP) and 1,3-propanediol(1,3-PDO), which are commercially attractive platform chemicals. Previous metabolic engineering of *K*. *pneumoniae* focused on the intensification of reduction pathway; however, it failed to overproduce 3-HP or 1,3-PDO. Contrary to this strategy, here we show that overexpression of glycerol dehydrogenase (dhaD), the first functional enzyme in oxidation pathway, can efficiently stimulate cell growth and facilitate 3-HP accumulation. Under microaerobic conditions, although metabolic burden arising from plasmid replication, the recombinant *K. pneumoniae* overexpressing *dhaD* grew actively and showed 60% enhancement of 3-HP compared to the control. In particular, overexpression of dhaD increased the activity of glycerol dehydratase, indicating the concerted action of two enzymes and the interdependence between glycerol oxidation and reduction pathways. Moreover, the strain overexpressing dhaD produced more lactic acid yet less acetic acid than the control, implying the interplay between dhaD expression and the formation of byproducts. Together, not only showing that intensifying glycerol oxidation pathway is beneficial to 3-HP production, this study also reveals the structural rigidity of *dha* operon that mediates glycerol dissimilation in *K*. *pneumoniae*.

## Introduction

**Figure 1.**
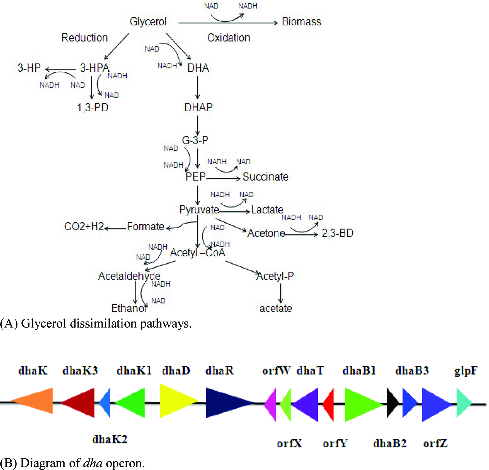
Diagram of glycerol dissimilation pathways and *dha* operon in *Klebsiella pneumonia*. (A) Glycerol dissimilation pathways. (B) Diagram of *dha* operon.

Biosynthesis has emerged as an alternative to chemical synthesis due to rapid depletion of fossil and petroleum resources. 3-Hydroxypropionic acid (3-HP) is a versatile platform compound, which can be readily changed into an array of bulk chemicals such as acrylic acid, 1,3-propanediol(1,3-PDO), malonic acid and polymers.[1–3] So far *Klebsiella pneumoniae* has been recognized as a promising host strain for production of 3-HP mainly because of its remarkable capacity to metabolize glycerol.[4–7] Under anaerobic or microaerobic conditions, *K. pneumoniae* can survive using glycerol as a sole carbon source (Figure 1(A)).[8] Glycerol dissimilation in *K*. *pneumoniae* is mediated by *dha* operon (Figure 1(B)), which involves parallel oxidation and reduction pathways.[8,9] In oxidation pathway, glycerol dehydrogenase (dhaD) catalyses glycerol into dihydroxyacetone (DHA), next, dihydroxyacetone kinase catalyses DHA into dihydroxyacetone phosphate (DHAP). DHA is the activator of *dha* operon. In reduction pathway, glycerol is converted to 3-hydroxypropaldehyde (3-HPA) by B_12_-dependent glycerol dehydratase (GDHt), 3-HPA is subsequently converted to 3-HP by aldehyde dehydrogenase (AldH), or to 1,3-PDO by 1,3-propanediol oxidoreductase.[10–12]

In contrast to glycerol oxidation pathway, reduction pathway has attracted more attention because it presents 3-HP and 1,3-PDO, which are in the list of 12 top valued chemicals proposed by US DOE.[3] To augment the metabolic flux to 3-HP or 1,3-PDO, previous metabolic engineering of *K. pneumoniae* focused upon the overexpression of enzymes in reductive pathway, or inactivation of enzymes in oxidative pathway. Unfortunately, through these strategies it is difficult to remarkably accumulate 3-HP or 1,3-PDO.[13,14] The explanation for this may be the structural rigidity of *dha* operon, which impedes preferential allocation of metabolic flux to any branched pathway. Given the close correlation between oxidation and reduction pathways, only intensification of reduction pathway cannot overproduce 3-HP.

Despite many reports concerning microbial conversion of glycerol to 3-HP, its commercialization is still in infancy.[15] One of hurdles hindering the production of 3-HP and 1,3-PDO lies in the slow cell proliferation.[7] Considering the close coupling between oxidation and reduction pathways, we reasoned that accelerating glycerol oxidation may be an efficient strategy for diverting flux to 3-HP. Thus, in this study, oxidation pathway instead of reduction pathway was intensified via plasmid-dependent overexpression of *dhaD* gene. Detailed analysis of glycerol consumption, cell growth, and the formation of 3-HP and byproducts could profoundly assess the influences of dhaD overexpression on *dha* operon. Together with previous work, this study aimed to provide a deeper understanding of *dha* operon and then propose a feasible strategy for production of 3-HP in *K. pneumoniae*.

## Materials and methods

### Strains, plasmids and reagents

*K*. *pneumoniae* DSM 2026 and *Escherichia coli* DH5α were strains obtained from DSMZ GmbH, Germany. The vector pET-28a (Novagen) was used in this study with minor modifications. The original T7 promoter was replaced by a native promoter *pk* of *dhaB1* gene, the first subunit of *dhaB* gene cluster (GenBank U30903) in *K. pneumoniae* DSM 2026. The resulting vector was designated as pET-pk. Restriction enzymes and Taq DNA polymerase were purchased from TaKaRa (Dalian, China). 3-HP was purchased from Tokyo Chemical Industry (TCI) Co. Ltd. (Tokyo, Japan). 1,3-PDO and other standard chemicals were products of Sigma. DNA synthesis and sequencing were performed by Beijing Sunbiotech Co. Ltd., China.

### Cultivation conditions

*E. coli* DH5α was grown in Luria–Bertani (LB) medium. The medium for producing 3-HP by *K. pneumoniae* contained the following components (per litre): K_2_HPO_4_·3H_2_O, 3.4 g; KH_2_PO_4_, 1.3 g; (NH_4_)_2_SO_4_, 4 g; MgSO_4_·7H_2_O, 0.5 g; CaCO_3_, 0.1 g; yeast extract, 3 g; glycerol, 40 g; and 1.25 mL of trace element solution. The trace element solution contained (per litre): ZnCl_2_·6H_2_O, 2.72 g; FeSO_4_, 32 g; MnCl_2_·4H_2_O, 0.68 g; CoCl_2_·6H_2_O, 1.88 g; H_3_BO_3_, 0.24 g; Na_2_MoO_4_, 0.02 g; CuCl_2_·2H_2_O, 1.88 g; and 40 mL of concentrated HCl. The recombinant strain was microaerobically grown in 50 mL Erlenmeyer's flask containing 25 mL medium with 50 μg/mL kanamycin at 37 °C and 120 *g* min^−1^ continuous shaking. The microaerobic condition was plugged by foam stopper.

### Construction of the recombinants

The *dhaD* gene encoding glycerol dehydrogenase was cloned by polymerase chain reaction (PCR) from the genomic DNA of *K. pneumoniae*. The native *pk* promoter of the gene *dhaB1* was used to drive gene expression. The 5′ and 3′ terminal DNA sequences of the *dhaD* gene (GenBank: NC_009648) were used to design the following primers: Forward: 5′-CGC*GGATCC*ATGCTAAAAGTTATTCAATCTCCAG-3′, Reverse: 5′-CCG*GAATTC*TTAACGCGCCAGCCACTGC-3′, where the underlined nucleotides indicate the *Bam*H I and *Eco*R I sites, respectively. The following is the PCR procedure: 94 ºC for 4 min; 94 ºC for 1 min, 55 ºC for 45 s, 72 ºC for 10 min, 30 cycles; 72 ºC for 10 min, 16 ºC hold. All other molecular manipulations followed the standard protocol.[16] The recombinant plasmids were transformed into *K. pneumoniae* DSM 2026, and the positive recombinants were screened by LB kanamycin plate and further identified by sequencing.

### Flask cultivation

The recombinant strains were grown in LB medium containing the following components per litre water: yeast extract 5 g, NaCl 10 g, peptone 10 g and kanamycin 50 mg. The recombinant strains were grown in 4 mL LB medium at 37 ºC for recovery. An amount of 1% of overnighted culture was inoculated to fermentation medium containing the same concentration of antibiotics. Microaerobic condition was achieved by using 250 mL Erlenmeyer flask containing 100 mL medium and shaking at 150 rpm, 37 ºC.

### Enzymatic activity assay

The activity of glycerol dehydratase (dhaB) in the crude cell extract was determined by a reported method.[17] The reaction mixture containing 35 mM potassium phosphate buffer (pH 8.0), 50 mM KCl, 50 mM glycerol and 15 μM coenzyme B_12_ was incubated at 37 ºC for 5 min before quenched by the addition of 100 mM citric acid. The 3-HPA formed in this reaction was dehydrated to acrolein, which was treated with tryptophan to form a purple complex. One unit of dhaB activity was defined as the amount of enzyme required to form 1 μmol of 3-HPA per minute.

The activity of glycerol dehydrogenase (dhaD) was determined by the method of Ahrens et al.,[18] where glycerol was used as the substrate and NAD^+^ as cofactor. One unit of dhaD activity was defined as the amount of enzyme required to change 1 μmol of NAD^+^ into NADH in one minute.

### Analytical method

Cell concentrations were measured by using microplate reader at 600 nm with 200 μL fermentation medium added in the cuvette. The metabolites 3-HP, lactic acid and acetic acid were measured by a high-performance liquid chromatography (HPLC) system (Shimazu, Kyoto, Japan) equipped with a C_18_ column and a SPD-20A UV detector. The mobile phase was 95% H_2_O, 5% methanol and 0.05% phosphoric acid at the flow rate of 0.8 ml/min. 1,3-PDO was measured by gas chromatography system (Shimazu, Kyoto, Japan). The used column was 2 m*ϕ5 mm, packed with chromsorb101, column temperature of 200 ºC, and detector and vaporizing chamber of 250 ºC. N_2_ was the carrier gas with a flow of 50 ml/min. All samples were filtered through a 0.22-μm membrane filter. The residual glycerol concentration was monitored every 3 h by a titration method with NaIO_4_ (for control of glycerol). All above experiments were performed in triplicate and subjected to SPSS 10.0 statistical software.

## Results and discussion

### Characterization of the recombinant strains

**Figure 2.**
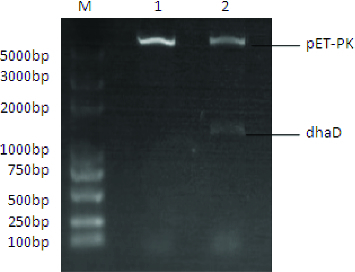
Restriction digestion of the control pET-pk and recombinant vector pET-pk-dhaD. M: DNA marker; 1: restriction digested blank vector; 2: digested recombinant vector.

Glycerol dissimilation in *K. pneumoniae* is split into reduction and oxidation pathways. Because dhaD catalyses the first committed step of oxidation pathway, it is the crucial enzyme for metabolic flux partition toward two branched pathways.[10] Hence, we investigated the effect of dhaD overexpression on 3-HP biosynthesis. The gene *dhaD* from *K. pneumoniae* was cloned by PCR. The recombinants Kp(pET-pk) and Kp(pET-pk-*dhaD*) were characterized by restriction digestion (Figure 2) and further confirmed by sequencing. The open reading frame of cloned *dhaD* gene was 1125 bp, which showed more than 98% homology with the reported sequence in the GeneBank, suggesting that it was correctly obtained.

### Effects of dhaD overexpression on glycerol consumption and cell growth

**Figure 3.**
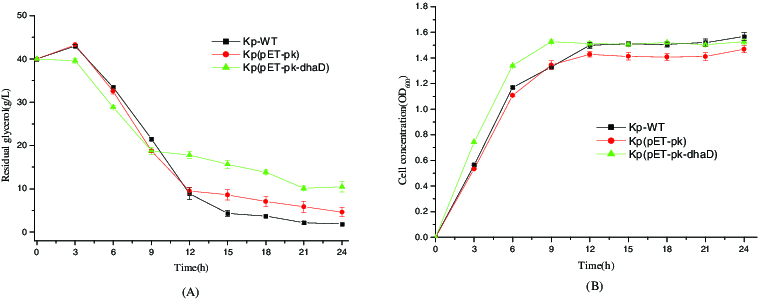
Glycerol consumption and cell growth of the wild-type and recombinant *K*. *pneumoniae*. (A) Residual glycerol levels in different strains detected at several time points. Kp-WT: wild-type *K. pneumoniae*; Kp(pET-pk): the recombinant *K. pneumoniae* harbouring blank vector pET-pk; Kp(pET-pk-*dhaD*): the recombinant *K. pneumoniae* harbouring *dhaD* gene in vector pET-pk. (B)Time curve of cell density in the same strains as described in (A) at corresponding time points. Error bars represent standard deviation from three independent experiments.

Overexpression of *dhaD* in this study aimed to accelerate glycerol oxidation and stimulate cell growth. As shown in Figure 2, in the first 9 h, all three strains including KP-WT (wild type *K. pneumoniae*), Kp(pET-pk) (the recombinant *K. pneumoniae* strain harbouring blank vector pET-pk) and Kp(pET-pk-*dhaD*) (the recombinant *K. pneumoniae* strain overexpressing *dhaD* gene) demonstrated similar patterns of glycerol consumption and cell growth, suggesting the obvious coupling between glycerol consumption and cell proliferation. With regard to glycerol consumption, in the first 9 h, the strain Kp(pET-pk-*dhaD*) consumed more glycerol than both Kp-WT and Kp(pET-pk) (Figure 3(A)), indicating that the overexpression of *dhaD* stimulated glycerol consumption (*P* ＜ 0.05). In accordance with glycerol consumption, Kp(pET-pk-*dhaD*) grew faster than two control strains during this period (Figure 3(B)), suggesting that *dhaD* overexpression facilitated cell proliferation. After 9 h, all three strains consumed glycerol slowly and entered stationary phase. In contrast to both Kp-WT and Kp(pET-pk), Kp(pET-pk-*dhaD*) consumed less glycerol but presented nearly equal biomass. Overall, if excluded the metabolic burden arising from plasmid replication, overexpression of *dhaD* actually benefited cell growth at initial phase of fermentation, but failed to maintain rapid growth during late phase of fermentation, implying the exquisite regulation of the *dha* operon.

### Effect of dhaD overexpression on byproducts formation

**Figure 4.**
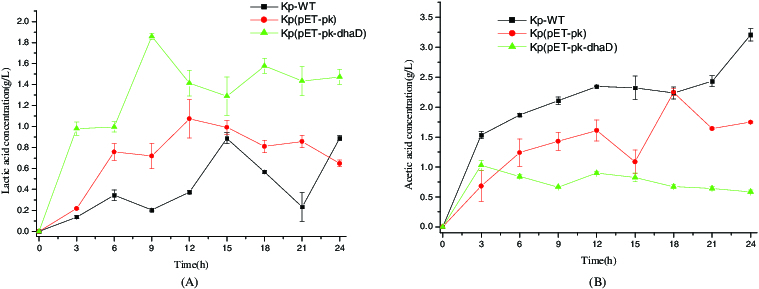
Byproducts produced by wild-type or recombinant *Klebsiella pneumoniae*. (A) Lactic acid production. (B) Acetic acid production. KP-WT: wild-type *K. pneumoniae*; Kp(pET-pk): the recombinant *K. pneumoniae* harbouring blank vector pET-pk; Kp(pET-pk-*dhaD*): the recombinant *K. pneumoniae* harbouring *dhaD* gene in vector pET-pk. The error bars represent standard deviation from three independent experiments.

Lactic acid and acetic acid are two major byproducts in the biosynthesis of 3-HP or 1,3-PDO. Their concentrations are shown in Figure 4. Compared to control strains KP-WT and Kp(pET-PK), Kp(pET*-pk-dhaD*) produced more lactic acid yet less acetic acid during a 24 h fermentation. In other words, overexpression of *dhaD* increased the carbon flux toward lactic acid but attenuated that toward acetic acid. This can be explained by the fact that overexpression of dhaD consumes NAD^+^ but simultaneously produces NADH, which is needed for the formation of lactic acid. Moreover, under anaerobic conditions, cell growth needs adenosine triphosphate (ATP) which is mostly provided by the formation of lactic acid. In contrast with acetic acid, lactic acid can be rapidly synthesized, because there is only one-step from lactic acid to pyruvate which is the hub of central metabolism. In contrast, the metabolic flux from pyruvate to acetic acid has to undergo three reactions. As shown in Figure 4, the recombinant produced small amount of acetic acid. In fact, attenuating the formation of acetic acid is beneficial to 3-HP production because acetic acid hinders cell growth due to cytotoxicity.[19,20]

### Effects of dhaD overexpression on 3-HP and 1,3-PDO production

**Figure 5.**
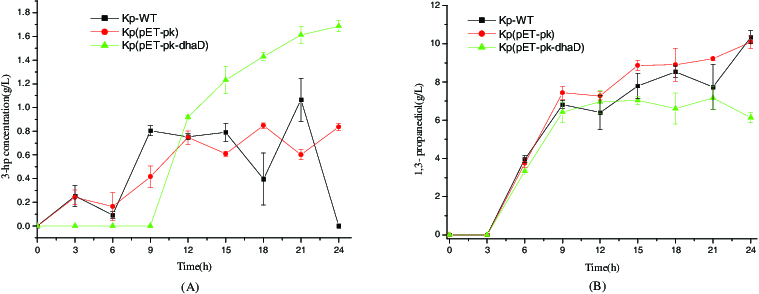
Metabolites produced in glycerol reductive pathway of different strains. (A) 3-hydroxypropionic acid. (B) 1,3-propanediol. KP-WT: wild-type *K. pneumoniae*; Kp(pET-pk): the recombinant *K. pneumoniae* harbouring blank vector pET-pk; Kp(pET-pk-*dhaD*): the recombinant *K. pneumoniae* harbouring *dhaD* gene in vector pET-pk. The error bars represent standard deviation from three independent experiments.

Since previous study has elucidated the close coupling between 3-HP formation and cell growth in log phase, we thereby reasoned that increasing the metabolic flux toward oxidation pathway is likely beneficial to 3-HP biosynthesis. To validate this deduction, *dhaD* gene was overexpressed. As depicted in Figure 5, from 15 to 24 h, the strain Kp(pET-pk-*dhaD*) produced more 3-HP yet less 1,3-PDO than both wild-type *K. pneumoniae* and that harbouring blank vector pET-pk. This result indicates a competition between 3-HP and 1,3-PDO for the metabolic flux from 3-HPA. One plausible explanation for this phenomenon is the generation and consumption of cofactors. As reported, 1,3-PDO synthesis depends on NADH availability.[21] Although dhaD overexpression is coupled with NADH production, 1,3-PDO biosynthesis ceased since 12 h because most metabolites in oxidation pathway also consumed NADH. In other words, there exists a competition for NADH among multiple reactions, and the generated NADP via dhaD overexpression is insufficient for 1,3-PDO production. Another likely reason for this may be the conversion of 3-HP to 3-HPA and next to 1,3-PDO, because these reactions are reversible. Collectively, the above results revealed the structural rigidity and flexibility of *dha* operon.

### Effect of dhaD overexpression on enzymatic activities

GDHt is the first enzyme in glycerol reduction pathway, while dhaD is the first enzyme in oxidation pathway. We thereby anticipate an inherent proportional relation between the two enzymes. To verify this deduction, we measured the activities of the two enzymes in strains Kp(pET-pk) and Kp(pET-pk-*dhaD*) that grew under microaerobic conditions in a shake flask. As shown in Table 1, both GDHt and dhaD in Kp(pET-pk-*dhaD*) exhibited higher activities (14.28 and 4.01, respectively) than those in the control strain Kp(pET-pk) (11.72 and 2.74), clearly indicating that dhaD overexpression increased the activities of GDHt and dhaD. This result reveals the intrinsic synergistic correlation between the two enzymes, which catalyse the committed reactions in two branched pathways. Presumably, when glycerol is the sole carbon source, the tailored *dha* operon entails a balanced partition of carbon flux toward oxidation and reduction pathways. And the synergistic activities of GDHt and dhaD contribute to this balance.

**Table 1.** The activities of glycerol dehydratase (GDHt) and glycerol dehydrogenase (dhaD) in recombinant strains Kp(pET-pk) and Kp(pET-pk-*dhaD*) grown under microaerobic conditions.

	Specific activity (U/mg)	
Strains	dhaD	dhaB (GDHt)
Kp(pET-pk)	2.74	11.72
Kp(pET-pk-*dhaD*)	4.01	14.28

Note: *Kp*(pET-pk): the recombinant *K. pneumoniae* harbouring blank vector pET-pk; Kp(pET-pk-dhaD): the recombinant *K. pneumoniae* harbouring *dhaD* gene in vector pET-pk.

Glycerol and glucose are two principal carbon sources for most if not all microorganisms. Under anaerobic conditions *K. pneumoniae* can survive using glycerol as sole carbon source. Glycerol dissimilation in *K. pneumoniae* is mediated by *dha* operon.[9] It seems that *dha* operon is tailored for the adaption to anaerobic environment when glucose had not been photosynthesized in the earth. As an independent machinery executing glycerol dissimilation, *dha* operon is supposed to be structurally rigid. In fact, the parallel glycerol oxidation and reduction pathways are interdependent branches and the intrinsic balance is hard to be broken. Presumably, the robustness and complexity of *dha* operon are due to the synergistic action of multiple events, including enzyme expression, cofactor consumption/regeneration, cell division and even quorum sensing. Previous metabolic engineering of *K. pneumoniae* failed to overproduce 3-HP or 1,3-PDO because the adopted strategies such as overexpression of key enzymes or regeneration of cofactor were inefficient to remodel the *dha* operon. Given the close coupling between cell growth and 3-HP synthesis, stimulating cell division may be an efficient strategy for accumulating 3-HP and 1,3-PDO.

## Conclusions

Instead of intensifying the glycerol reduction pathway, in this study, the oxidation pathway was augmented by overexpressing its first crucial enzyme – dhaD. Under microaerobic fermentation conditions, the recombinant *K. pneumoniae* overexpressing *dhaD* showed high GDHt activity and active cell growth (0–12 h) compared to the wild-type *K. pneumoniae* and to the harbouring blank vector pET-pk, indicating the synergistic action of dhaD and GDHt and thus the close coupling between glycerol oxidation and reduction pathways. Additionally, the strain Kp(pET-pk-*dhaD*) produced more 3-HP and lactic acid, yet less 1,3-PDO and acetic acid than the control, reflecting the sophisticated interplay between cofactor metabolism and 3-HP/1,3-PDO synthesis. Not only suggesting that intensifying the oxidation pathway is a feasible strategy for accumulating 3-HP, this study also sheds light on the structural rigidity of *dha* operon in *K. pneumoniae*. Overall, this work may enlighten the upcoming biological production of 3-HP and 1,3-PDO.
